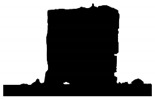# Improvement in Char Strength with an Open Cage Silsesquioxane Flame Retardant

**DOI:** 10.3390/ma10060567

**Published:** 2017-05-23

**Authors:** Yolanda Bautista, Ana Gozalbo, Sergio Mestre, Vicente Sanz

**Affiliations:** Instituto Universitario de Tecnología Cerámica, Universitat Jaume I, 12006 Castellón, Spain; ana.gozalbo@itc.uji.es (A.G.); sergio.mestre@itc.uji.es (S.M.); sanzs@qui.uji.es (V.S.)

**Keywords:** 3-methacryloxypropyltrimethoxysilane (MAPTMS), open silsesquioxane, hydrogen bond, fire retardance, thermal degradation

## Abstract

Different characterization techniques were used to study the hydrolysis and condensation reaction kinetics of 3-methacryloxypropyltrimethoxysilane (MAPTMS) to obtain open cage silsesquioxane oligomers. The formation of hydrogen bonds, which condition the chemical structures of the resulting products, was identified. Improved thermal and fire resistant behavior of unsaturated polyester (UP) composites prepared with aluminium trihydroxide (ATH) and the synthesized oligomer were registered. Opened silsesquioxane structures also showed an improvement in the mechanical properties of the char formed after firing.

## 1. Introduction

Unsaturated polyester (UP) resins are used in numerous applications, such as vehicle bodies, ship hulls, encapsulated electric components, and solid surface composite materials. Polyesters are flammable materials and, in the presence of the oxygen in air, the flame propagates spontaneously. In fire situations, dropping may occur while burning which may start secondary fires. To delay or prevent the fire from spreading, flame retardants are added and dispersed in polymers. The nature of these additives (halides, phosphates, hydroxides, aluminosilicates, etc.), which act by different physical and/or chemical mechanisms in the fire propagation phases, varies greatly [1,2].

Inorganic polymers such as silicones or polysiloxanes exhibit greater heat resistance than organic polymers and are widely used as lubricants, coatings, and adhesives in systems subject to high working temperatures. This greater thermal stability stems from the presence of Si–O type bonds, which have higher binding energies (about 461 kJ mol^−1^) than the C–O (358 kJ mol^−1^) and C–C (304 kJ mol^−1^) bonds in organic polymers [3]. Therefore, the incorporation of polyhedral oligomeric silsesquioxanes (POSS) into polymeric materials or composites improves mechanical properties, thermal stability, and fire resistance [4]. Much attention has been focused on the use of closed cage POSS structures in the literature, ignoring the potential of open cage structures.

In the present study, organic–inorganic hybrid oligomers with silsesquioxane (SiO_1.5_) bonds in open cage structures were synthesized and polymerized together with unsaturated polyester resins and aluminium trihydroxide (ATH), a classic flame retardant. The materials obtained exhibited significantly increased thermal stability, fire resistance, and improved mechanical resistance after firing due to the open cage structures. The silsesquioxane oligomer was also structurally characterized.

## 2. Results and Discussion

### 2.1. Silsesquioxane Oligomers

The organic–inorganic hybrid oligomers were synthesised by the sol–gel method from 3-methacryloxypropyltrimethoxysilane (MAPTMS) with hydrolysable methoxy groups [5], which allowed formation of siloxane bonds under practically ambient conditions [6]. The hydrolysis and condensation reaction kinetics depend on variables such as the H_2_O:Si mole ratio, solvent nature, pH, temperature, and steric hindrance of the silanes, as well as if they are catalysed in acid and basic media [6]. Oligomers with different degrees of hydrolysis and condensation can be obtained as a function of the different reaction conditions.

In general, these oligomers, known as POSS, have a unit that is repeated with the formula RSiO_3/2_. The term ‘silsesqui’ refers to the Si:O atom ratio, which is 1:1.5. A silsesquioxane type oligomer can exhibit different molecular structures. The open structures may be partially condensed, whereas the closed ones are always fully condensed. The closed structures, with a prism shape, may have a triangular, square, pentagonal, or hexagonal base [7]. In POSS involving polyhedrons composed of silicon and oxygen atoms, the silicon atoms are located at the vertices of the polyhedron and the edges are formed by Si–O–Si bonds. The organic substituents of the silicon trialkoxides are oriented towards the outside of the polyhedron, linked through the silicon vertices.

**Figure 1:**
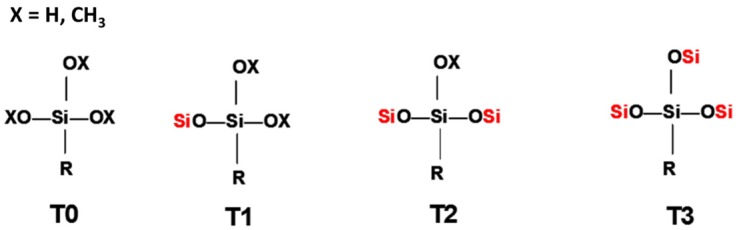
Scheme of the nomenclature used to define the degree of condensation of the trialkoxysilanes [8].

In order to identify the different silicon atoms, present in the oligomer synthesised in the present study, the conventional Tn nomenclature proposed by Engelhardt will be used hereinafter [8]. The letter T stands for the silicon atoms with three potential hydroxyl or alkoxy reactive groups. The symbol ‘n’ stands for the number of other silicon atoms to which the first silicon is linked to through bridging oxygen atoms (Figure 1).

**Figure 2:**
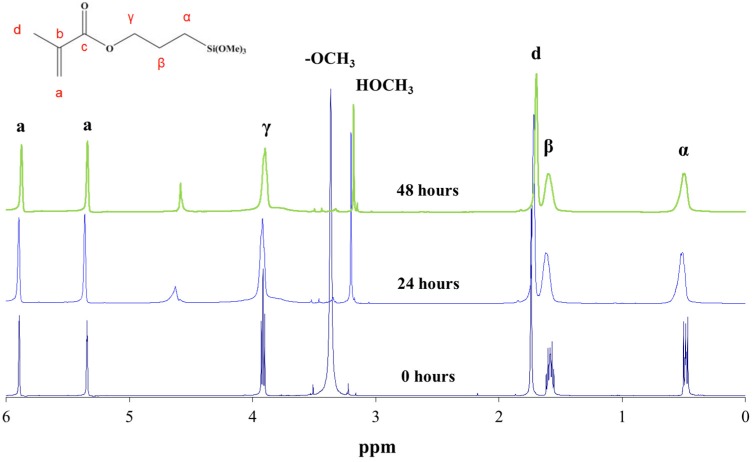
^1^H NMR spectrum of 3-methacryloxypropyltrimethoxysilane (MAPTMS) at 0, 24, and 48 h of reaction.

The hydrolysis reaction progress of MAPTMS, performed under acid catalytic conditions, was monitored by ^1^H NMR spectroscopy. The protons of the MAPTMS molecule, labelled with different letters, and their signals in the spectra corresponding to the starting samples and after 24 h and 48 h reaction, are shown in Figure 2. 

The spectrum of the sample after the 24 h reaction exhibited a drastic decrease in the methoxy group (OCH_3_) signal, which was reduced to a set of signals that disappeared into the noise. This behaviour indicates that the hydrolysis reaction took place in the first few hours of the reaction [9], because in acid medium the hydrolysis rate is usually faster than the condensation rate [6]. In fact, in all the reacted samples spectra, the signal corresponding to the methoxy groups of methanol, which was generated as a by-product of the hydrolysis reaction, appeared at 3.18 ppm. The hydroxyl group (OH) bonded to the silicon after the hydrolysis reaction could correspond to the signal appearing at 4.63 ppm. 

In the unreacted MAPTMS spectrum, the α, β, and γ proton signals split due to coupling with vicinal protons. The hydrolysis and condensation reactions produced numerous chemical species. The chemical shift of the α, β, and γ protons of these species changed slightly, producing very broad peaks.

**Figure 3:**
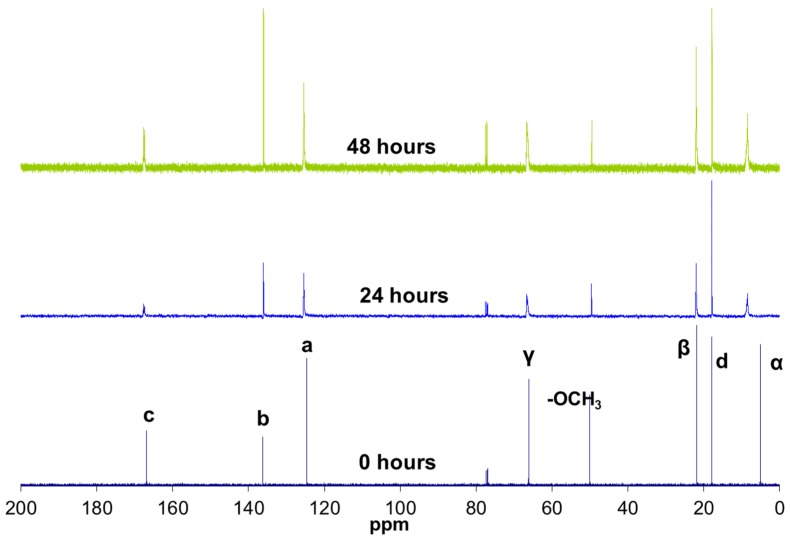
^13^C NMR spectrum of MAPTMS at 0, 24, and 48 h of reaction.

The oligomer chemical structure was monitored also by ^13^C NMR spectroscopy (Figure 3). This information allowed the reaction kinetics to be subsequently understood. Two major types of changes were observed in these spectra with the reaction progress: changes in the chemical shifts and in the duplications (Table 1).

On the one hand, the carbon signals shifted towards lower fields (left side of the spectrum). This shift was very significant in the case of the α carbons, which were nearest to the reactive silicons. In the reactions, the methoxy groups (Si–OCH_3_) were replaced with silsesquioxane groups (Si–O–Si) with lower electron affinity. The carbons near the silicon therefore exhibited greater electron density and resonated at lower fields.

On the other hand, in addition to shifting, the signals of the a, c, and γ carbons (close to the carbonyl group) were duplicated in two peaks. This duplication might be due to the presence of hydrogen bonds between a fraction of the methacrylate carbonyl groups (C=O) and a hydroxyl group (OH) bonded to the silicon after the hydrolysis reaction. The hydrogen bonds decreased the carbon electron density of the carbonyl group, thus resonating at lower frequencies. The intensities observed in the duplicated peaks indicated that the carbonyl group fraction with hydrogen bonds was 0.35.

**Figure 4:**
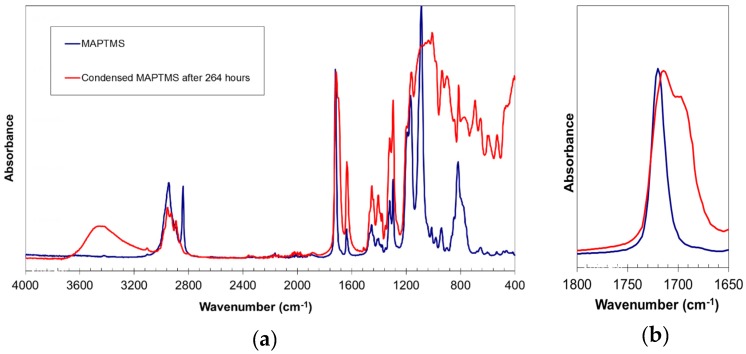
FT-IR spectrum of MAPTMS and of the MAPTMS hydrolysis and condensation product after 264 h of reaction, (**a**) complete spectra, (**b**) details of the carbonyl signals.

The literature reports that infrared spectroscopy can be used to assess the presence of hydrogen bonds in various silane condensation products, such as methacryloxypropyltrimethoxysilane [10], poly(acrylic acid)/silica hybrid [11], and tetraethoxysilane [12]. In the present study, infrared spectroscopy also confirmed the presence of hydrogen bonds (Figure 4). The reaction product exhibited a very broad band centred at 3460 cm^−1^, corresponding to the O–H bond vibration. The broadness of this band indicated that most of the hydroxyl groups are participating in hydrogen bonds. The appearance of a shoulder at 1703 cm^−1^ next to the carbonyl group C=O vibration band (1716 cm^−1^) is usually associated with the presence of hydrogen bonds [10]. Hydrogen bonds lengthen and weaken the C=O bond, leading to absorption at lower frequencies [11,12].

**Figure 5:**
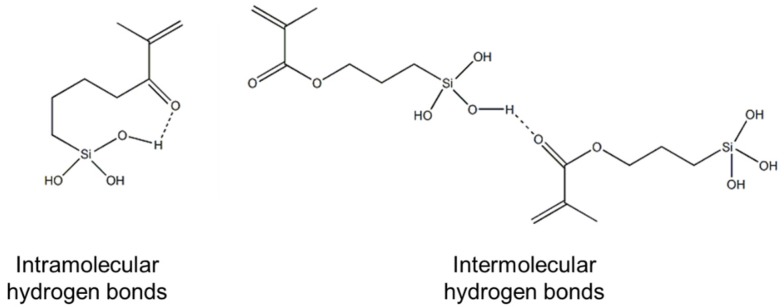
Chemical structure of the intramolecular and intermolecular hydrogen bonds.

The hydrogen bonds identified by ^13^C NMR and FT-IR could be either intramolecular or intermolecular interactions, or combinations of both, as illustrated in Figure 5; however, these techniques do not allow us to differentiate between the two types of interactions.

**Figure 6:**
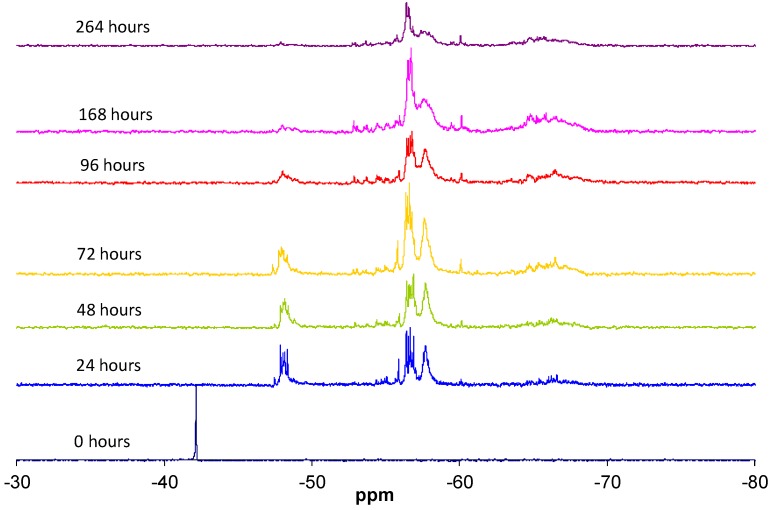
^29^Si NMR spectra of MAPTMS at different reaction times.

The condensation reaction progress was monitored by NMR spectroscopy of the ^29^Si nucleus (Figure 6). According to the literature [13], the chemical shifts of ^29^Si for trialkoxysilanes are located in the range from −35 to −75 ppm. Each alkoxide condensation reaction causes the ^29^Si signal to shift by about 8–9 ppm towards higher fields in the spectrum, allowing four large areas to be identified, corresponding to the different degrees of condensation; T0, T1, T2, and T3 (Figure 1).

The replacement of an alkoxide group (OR) with a hydroxyl group (OH) modifies the chemical shift of ^29^Si between 0.4 and 0.8 ppm [13]. The progress of the hydrolysis and condensation reactions increases the number of combinations of the silicon atom chemical environments, which is reflected in a complex structure of signals in each of the four areas of the spectrum [14].

It was taken into account that the participation of the silicon atom in small cycles reduces the angle of the Si–O–Si bond with relation to the linear species, inducing a signal shift towards lower fields (left of the spectrum). That is, in the T2 signal area, the silicons included in cycles are shifted to the left [15].

The data reported in the literature enabled the groups of signals in the ^29^Si NMR spectrum, detailed in Table 2, to be identified. The subscripts used indicate the number of hydroxyl groups still present, while the reference in brackets indicates the linear (l), branched (r), or cyclical (c) structure and the number of silicon units forming the structure.

**Figure 7:**
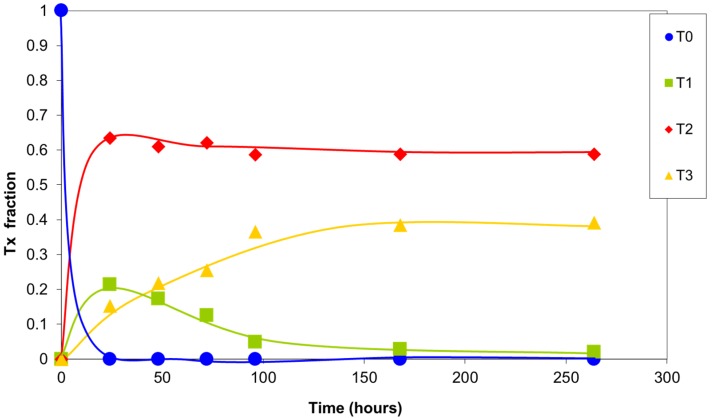
Relative concentration of the types of silicon atoms in the sol–gel reaction products with time. Values of the integrals obtained in the ^29^Si NMR spectrum.

The integrals of the signals of silicon T0, T1, T2, and T3 are plotted versus reaction time in Figure 7. In the first 24 h, the highly reactive T0 silicons were completely consumed, forming structures with a greater degree of condensation. The T1 silicon formed rapidly, exhibiting a maximum concentration at 24 h of the reaction. Their concentration then progressively decreased due to condensation, giving rise to T2 and T3 silicon. A large number of T2 silicons were present after the first few hours of the reaction, with a maximum at 24 h of the reaction. Their concentration then decreased slightly as a result of the balance between the newly formed T2 silicons and those that were transformed into T3 silicons. The T3 silicon concentration increased continuously due to the reaction of less condensed silicons. Finally, under the condensation conditions used, a distribution of T2 and T3 silicons was attained that depended on the final structures that formed. 

The reactivity of the silicon species generally decreased as their degree of condensation increased, as had already been observed by other researchers [16,17].

**Figure 8:**
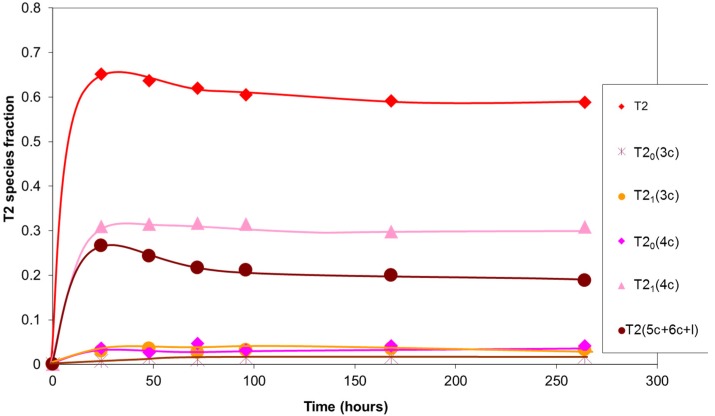
Evolution with time of the fraction of T2 type silicon atoms in the sol–gel reaction products. Values of the integrals obtained in the ^29^Si NMR spectrum.

Further analysis of T2 silicons (Figure 8) revealed that about half of these participated in tetramer cycles with a hydroxyl group (T2_1_(4c)), with their concentration remaining constant over time. The sum of the T2 silicons that formed part of the pentamer and hexamer cycles and linear species (T2(5c+6c+l)) was of the same order, though it decreased with time. The decrease might be due to the transformation of the linear species into cyclical species. This transformation of the linear species in acid medium has been reported in the condensation reactions of other silanes, such as methyltrimethoxysilane [16], methyltriethoxysilane [15], and tetraethoxysilane [18]. The presence of T2 silicons in trimer cycles was very small. 

The stability of T2_1_(4c) silicons might be explained by the existence of hydrogen bonds, described above, which could inhibit the nucleophilic attack of the condensation reaction. The T2_1_(4c) silicon fraction identified with respect to the total existing silicons was about 0.30. This value was very similar to that of the carbonyl group fraction affected by hydrogen bonds, identified by ^13^C NMR, which reinforces the role of these bonds in hindering silicon atom reactivity.

**Figure 9:**
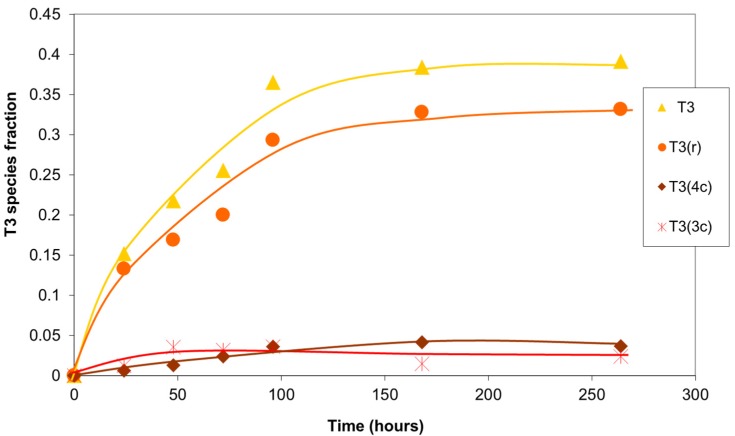
Evolution with time of the fraction of T3 silicon atoms in the sol–gel reaction products. Values of the integrals obtained in the ^29^Si NMR spectrum.

Analysis of the T3 silicons (Figure 9) revealed a minor presence of prismatic structures (T3(4c) and T3(3c)). Most of the fully condensed silicons formed part of the branched structures, T3(r), less subject to the stresses of several cycles. These results were observed, with minor variations, for the different batches of the oligomer prepared.

Hydrolysis and condensation in the sol–gel process give rise to numerous species, generically known as silsesquioxanes, having the general formula: T_n_(OH)_x_(OCH_3_)_y_, where T = RSiO_1.5-m/2n_, with (m = x + y), according to the nomenclature used by Eisenberg et al. [19]. The molecular masses of the chemical species arising as a result of MAPTMS hydrolysis and condensation were quantified by mass spectrometry. Data analysis then allowed the main chemical structures to be identified.

**Figure 10:**
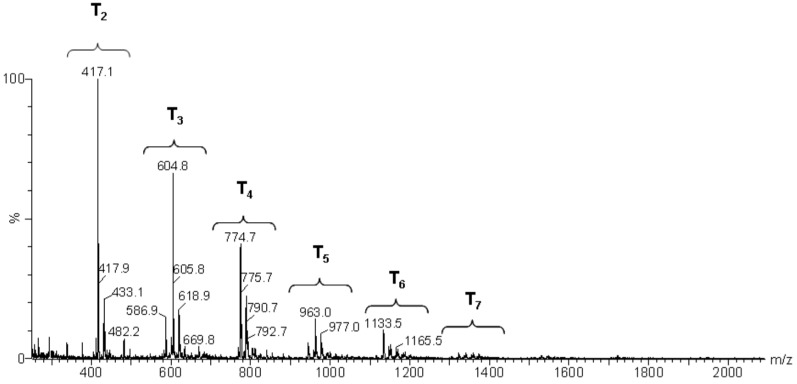
Electrospray ionization time-of-flight mass spectrum (ESI–TOF MS) of the MAPTMS hydrolysis and condensation product after 24 h of reaction.

**Figure 11:**
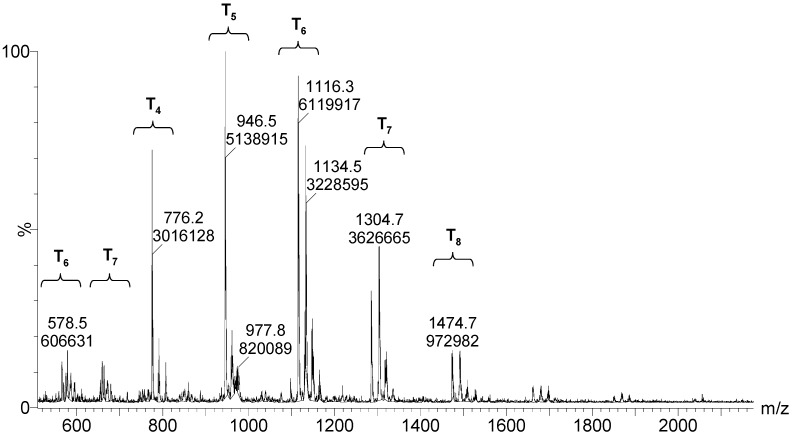
ESI–TOF MS of the MAPTMS hydrolysis and condensation product after 264 h of reaction.

The mass spectra after 24 h and 264 h of the reaction (Figure 10 and Figure 11, respectively) displayed a primary distribution of the species, corresponding to the number of bonded monomers (n = 2 dimers; n = 3 trimers; n = 4 tetramers, etc.). At the same value of n, a secondary distribution corresponding to the variation of m, by modification of both the value of x and the value of y was observed. In the secondary distribution, the value of m indicated the number of intramolecular cycles, while the value of y indicated the presence of unhydrolyzed alkoxide groups.

Mass spectroscopy showed that, after 24 h of reaction, no independent silane molecules (monomers T1) remained. The existing oligomers consisted of 2 to 7 silsesquioxane molecules. Table 3 lists the major species, corresponding to about 68% by weight of the sample. They were mainly linear dimer and trimer structures, in addition to single-cycle tetramers. Although most were species with fully hydrolysed alkoxide groups, some oligomers still had at least one unhydrolyzed alkoxide group. Table 3 includes a schematic illustration of the possible structures for each mass/charge ratio: the black dots represent the silicon atoms, while the segments between two black dots represent the links between silicon atoms through oxygen atoms, i.e., Si–O–Si type bonds. The species with an unhydrolyzed group have not been included for the sake of simplicity. However, these would be similar to those illustrated, with one of the OH groups being replaced with an OCH_3_ group.

After 264 h of the reaction, the least condensed oligomers were the tetramers (n = 4). Table 4 lists the major species, corresponding to about 68% by weight of the sample. These were oligomers obtained by condensation of four to eight silane molecules, with a high number of intramolecular cycles and complete hydrolysis of the alkoxide groups. The table includes a schematic illustration of the possible structures for each mass/charge ratio.

The structural analysis above indicates that the oligomers consisted mainly of four to eight silsesquioxane molecules containing unreacted hydroxyl groups (hydrolysed silanes but not yet condensated). The participation of these groups in hydrogen bonds with the methacrylate groups prevented the condensation reactions from concluding. The oligomers exhibited a high number of cycles per molecule without, however, forming the closed polyhedral structures characteristic of POSS. 

### 2.2. Unsaturated Polyester Composite Materials

Different combinations of fire retardant components were introduced in the UP composite in order to study the synergy effect between the aluminium trihydroxide and silsesquioxane oligomers (Table 5). In all cases, the total filler content was 60% w/w. 

The effectivity of aluminium trihydroxide as a fire retardant for polymers is related to a high load percentage. When 60 wt % ATH was incorporated into the pure UP resin, the limiting oxygen index (LOI) was significantly increased from 21% to 44%, even though the combustion residue had not enough mechanical resistance to be manipulated. The effect on the fire behaviour of the partial substitution of 5 wt % of ATH for silsesquioxane with different reaction times and consequently different condensation degrees was evaluated. A further increase in the LOI value (up to 50%) was observed with the condensation time until 48 h of reaction time, the increase being significant as only 5% of ATH had been substituted by silsesquioxane. Similar LOI values were detected for a commercial completely condensed POSS (R40A55P5). Therefore, a complementary effect between aluminium trihydroxide and silsesquioxane fire retardants was observed.

**Figure 12:**
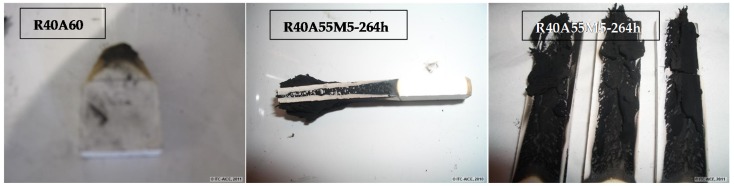
Combustion residues obtained from the limiting oxygen index (LOI) measurement.

A different behaviour was also observed with the char strength. The composite prepared with only ATH as the fire retardant produced char that did not support their own weight (Figure 12). However, the composites prepared with a combination of ATH and silsesquioxane oligomer form char with enough consistency to measure their mechanical resistance. As showed in Table 5, the mechanical resistance of the combustion residue of composites with open cage silsesquioxanes was increased with the condensation reaction time. This behaviour was not correlated with the full polyhedral condensated methacryl POSS. Thus, the improvement of the mechanical properties of the combustion residue would be related to highly condensed silsesquioxane oligomers in open polyhedral structures. The presence of open-cage silsesquioxane oligomers in composites allowed interactions between components during firing that improved the mechanical properties of the char formed and avoided dropping, as was verified by hot stage microscopy (Table 6).

**Figure 13:**
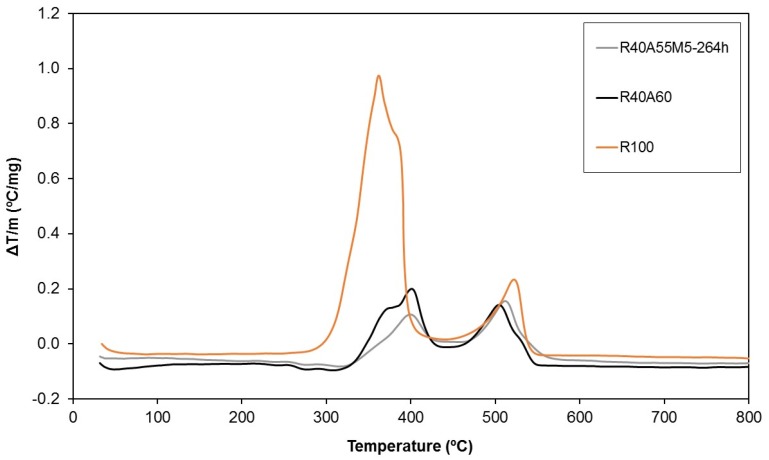
Differential thermal analyses of three of the studied samples.

The thermal stability of the UP composites was also evaluated by differential thermal analysis (DTA) (Figure 13). Although highly exothermic complex combustion reactions of the pure polyester polymer (R100) were registered between 300 °C and 400 °C, a noteworthy decrease of the exothermic character was observed by the introduction of aluminium trihydroxide (R40A60). The improvement in the thermal behaviour of the composites was slightly increased by the combination of silsesquioxanes and aluminium trihydroxide as fire retardants (R40A55M5-264h).

## 3. Materials and Methods

### 3.1. Materials

3-Methacryloxypropyltrimethoxysilane (MAPTMS) (Sigma-Aldrich Química, S.A, Madrid, Spain), was selected as the precursor of the silsesquioxane oligomer, as it has reactive unsaturations in the methacrylate group. Methacryl POSS was supplied by Hybrid Plastics Inc. (Hattiesburg, MS, USA). Aluminium trihydroxide (ATH) was kindly supplied by Huber Engineered Materials (Ostende, Belgium).

Unsaturated polyester resin was kindly supplied by Ferro Spain, S.A. (Castellon, Spain), (obtained by the reaction between maleic anhydride, phthalic anhydride, and ethylene glycol, and later diluted in styrene). Accelerator NL-51P, a cobalt(II) 2-ethylhexanoate 6% wt Co, in a solvent mixture, was employed as an accelerator, and Butanox LA, a methyl ethyl ketone peroxide (MEKP) solution, as an initiator. Both additives were supplied by Akzo Nobel Chemicals S.A. (El Prat de Llobregat, Spain).

### 3.2. Preparation of Silsesquioxane Oligomers

The synthesis was performed under acid-catalysed conditions since, according to the literature [6], condensation yields smaller-sized molecules than those obtained under basic conditions. In a typical synthesis, 20 g (0.08 moles) of MAPTMS was poured into a 200 mL vessel. The silane was stirred while 6.5 g (0.36 moles) of a 0.01 N HCl aqueous solution was slowly added. Then, the synthesis was performed with a H_2_O:Si mole ratio = 4.5:1 because this was the ratio that led to more condensed structures. The mixture was constantly stirred. Materials were extracted at reaction times of 24 h, 48 h, 72 h, 96 h, 168 h, and 264 h. The solvent was evaporated off under vacuum and a clear viscous product was obtained.

### 3.3. Preparation of Polymer Composites

The unsaturated polyester resin was previously activated with 1% of Accelerator NL-51P. The polymer composites were prepared by mixing 40 wt % of activated unsaturated polyester resin with 60 wt % of a fire-retardant mixture. Different combinations of fire retardants were tested. The polymerization reaction was initiated by adding 2% of Butanox LA and then the mixture was cast into 20 mm × 80 mm × 6 mm moulds. 

### 3.4. Silsesquioxane Oligomer Structural Characterisation

The MAPTMS hydrolysis and condensation reactions were monitored by nuclear magnetic resonance (NMR) spectroscopy of the ^1^H, ^13^C, and ^29^Si nuclei. This technique allowed the different degrees of silsesquioxane condensation during the reaction to be quantified. A Varian VNMR System 500 MHz spectrometer (Varian Inc , Palo Alto, CA, USA) was used. The chemical shifts were expressed in parts per million (δ ppm) with respect to tetramethylsilane (TMS). Deuterated chloroform (CDCl_3_) was used as an internal reference. 

The study of the chemical species present in the synthesised oligomer was completed by Fourier transform infrared spectroscopy (FTIR) and mass spectrometry. The infrared spectra were obtained on a THERMO-NICOLET 6700 instrument (Thermo Fischer Scientific Inc., Waltham, Massachusetts), between 400 and 4000 cm^−1^, recording 16 scans at 4 cm^−1^ resolution. The electrospray ionization time-of-flight mass spectra (ESI-TOF-MS) were obtained on a QTOF I (quadrupole–hexapole–TOF) hybrid spectrometer (Micromass UK Ltd., Wilmslow, UK) with a Z-spray electrospray orthogonal interface.

### 3.5. Polymer Composite Characterisation

DTA was used in order to study the thermal resistance of the polymer and composite materials. The DTA measurements were performed with a TGA/SDTA 851e thermal analysis apparatus (Mettler Toledo International Inc., L'Hospitalet de Llobregat, Spain) between 30 °C and 800 °C with a heating rate of 5 °C/min under air. Around 15 mg of samples were introduced in platinum crucibles, with ignited alumina (Al_2_O_3_) chosen as the reference material. The evolution of the shape of the specimens during firing was evaluated by hot stage microscopy. The experiments were run on a MISURA 2 (Expert System Solutions, Modena, Italy) with a heating rate of 25 °C/min. Images of the specimen silhouettes were taken every 10 °C. 

The LOI was obtained following the ISO 4589:2001 procedure, which involves measuring the minimum oxygen concentration required for self-sustained combustion of the samples [20]. The mechanical strength of the combustion residue was determined from prism-shaped test 6 mm × 20 mm × 80 mm pieces obtained after firing in LOI testing. The bending strength was determined by the three-point bending method with an universal testing machine 6027 (Instron, Norwood, MA, USA).

## 4. Conclusions

Silsesquioxane oligomers were synthesised in open structures by the hydrolysis and condensation reactions of MAPTMS. Different characterisation techniques were used to study the arising molecular structures. The presence of hydrogen bonds between the carbonyl groups and the hydroxyls was identified by both NMR and FT-IR spectroscopy and could be hindering the closed-cage structures.

A complementary effect was observed between aluminium trihydroxide and silsesquioxanes in the thermal and fire retardant properties of the UP composites. Firstly, the exothermicity of the combustion reaction was decreased. Secondly, the minimum oxygen concentration required to sustain combustion (LOI) was significantly increased. Furthermore, obtaining open silsesquioxane structures instead of closed cage structures also improved the mechanical properties of the char obtained after firing.

## Figures and Tables

**Table 1:** The most significant changes and duplications of the ^13^C NMR chemical shifts at three the reaction times.

Reference	Reaction Time				
	*t* = 0 h	*t* = 24 h		*t* = 48 h	
	Chemical Shifts (ppm)	Chemical Shifts (ppm)	Intensity Ratio	Chemical Shifts (ppm)	Intensity Ratio
α	5.07	8.50	---	8.50	---
γ	66.16	66.38 66.68	0.40 0.60	66.36 66.65	0.35 0.65
a	124.64	125.31 125.44	0.32 0.68	125.29 125.42	0.34 0.66
c	166.85	167.43 167.67	0.32 0.68	167.34 167.61	0.34 0.66

**Table 2:** Band assignment in the ^29^Si NMR spectra of the MAPTMS reaction.

Species	Chemical Band Shift (ppm)
T1	−47.4 to −49.3
T2_1_(3c)	−52.7 to −54.2
T2_0_(3c)	−54.2 to −55.4
T2_1_(4c)	−55.4 to −56.1
T2_0_(4c)	−56.1 to −57.3
T2(5c+6c+l)	−57.3 to −58.1
T3(3c)	− 58.1 to −59.2
T3(4c)	−59.2 to −60.7
T3(r)	−62 to −69

**Table 3:** Summary of the major species detected in the oligomer by mass spectrometry after 24 h of reaction.

Experimental m/z Value	Integration	Species	Structures
417.1	20%	T_2_(OH)_4_Na^+^ (l)	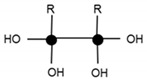
433.1	6%	T_2_(OH)_3_(OCH_3_)Na^+^ (l)	
604.8	15%	T_3_(OH)_5_Na^+^ (l)	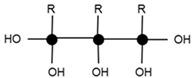
618.9	8%	T_3_(OH)_4_(OCH_3_)Na^+^ (l)	
774.7	11%	T_4_(OH)_4_Na^+^ (c)	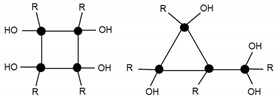
788.7	8%	T_4_(OH)_3_(OCH_3_)Na^+^ (c)	

**Table 4:** Summary of the major species detected in the oligomer by mass spectrometry after 264 h of reaction.

Experimental m/z Value	Integration	Species	Structures
1115.3	15%	T_6_(OH)_2_Na^+^ (3c)	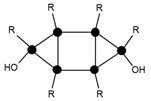
945.5	13%	T_5_(OH)_3_Na^+^ (2c)	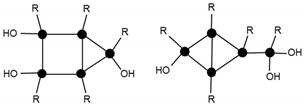
664.3 1303.7	9%	T_7_(OH)_3_Na_2_^+2^ (3c) T_7_(OH)_3_Na^+^ (3c)	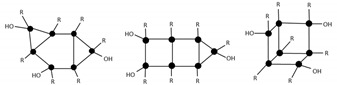
578.5 1133.5	9%	T_6_(OH)_4_Na_2_^+2^ (2c) T_6_(OH)_4_Na_2_^+2^ (2c)	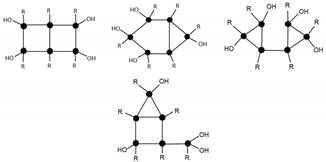
775.2	7%	T_4_(OH)_4_Na^+^ (c)	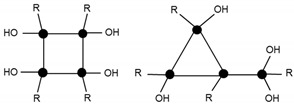
55.6 1285.0	6%	T_7_(OH)Na_2_^+2^ (4c) T_7_(OH)Na (4c)	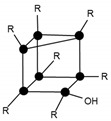
587.0 1149.4	5%	T_6_(OH)_6_Na_2_^+2^ (1c) T_6_(OH)_6_Na^+^ (1c)	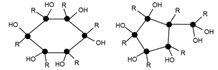
1491.2	4%	T_8_(OH)_4_Na^+^ (3c)	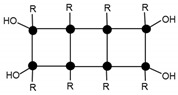

**Table 5:** Compositions of the unsaturated polyester (UP) composites and fire retardant behavior.

Composite	Resin	ATH	MAPTMS (Synthesis Time)	POSS	Limiting Oxygen Index (LOI)	Mechanical Resistance of Combustion Char (kg/cm^2^)
R100	100%	0%	0%	0%	21%	---
R40A60	40%	60%	0%	0%	44%	---
R40A55M5-0 h	40%	55%	5% (0 h)	0%	46%	0.7 ± 0.3
R40A55M5-24 h	40%	55%	5% (24 h)	0%	48%	0.7 ± 0.2
R40A55M5-48 h	40%	55%	5% (48 h)	0%	50%	1.2 ± 0.1
R40A55M5-96 h	40%	55%	5% (96 h)	0%	50%	2.3 ± 0.2
R40A55M5-264 h	40%	55%	5% (264 h)	0%	50%	2.8 ± 0.2
R40A55P5	40%	55%	0%	5%	51%	0.8 ± 0.1

**Table 6:** Silhouettes of the samples during the hot stage microscopy measurements.

Temperature (°C)	R100	R + M
25	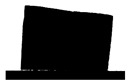	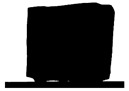
350	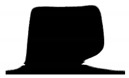	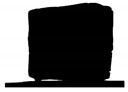
380	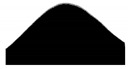	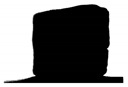
400	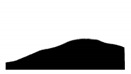	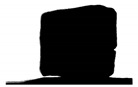
800	---